# Associations between *TNF* gene promoter variants (rs361525, rs1800629, rs1799964, rs1799724) and the clinical course of idiopathic inflammatory myopathies

**DOI:** 10.1038/s41598-025-25258-z

**Published:** 2025-11-21

**Authors:** Agnieszka Padjas, Anna Mikołajczyk-Korona, Radosław Dziedzic, Sylwia Dziedzina, Marek Sanak, Stanisława Bazan-Socha, Jan G. Bazan, Lech Zaręba, Mariusz Korkosz, Joanna Kosałka-Węgiel

**Affiliations:** 1 Department of Internal Medicine, Faculty of Medicine, Jagiellonian University Medical College, Jakubowskiego 2, 30-688 Kraków, Poland; 2 Doctoral School of Medical and Health Sciences, Jagiellonian University Medical College, św. Łazarza 16, 31-530 Kraków, Poland; 3 Faculty of Exact and Technical Science, Institute of Computer Science, University of Rzeszów, Pigonia 1, 35-310 Rzeszów, Poland; 4 Department of Rheumatology and Immunology, Jagiellonian University Medical College, Jakubowskiego 2, 30-688 Kraków, Poland

**Keywords:** Idiopathic inflammatory myopathies, Tumor necrosis factor, *TNF* gene, Genetics, Immunology, Rheumatology

## Abstract

Idiopathic inflammatory myopathies (IIM) are characterized by muscle weakness and immune-mediated muscle injury. Tumor necrosis factor (TNF) appears to be involved in the pathogenesis of IIM, especially regarding susceptibility to malignancy. Here, we analyzed associations between variants of the *TNF* gene promoter and the clinical course of the disease. We conducted a case–control study that analyzed selected variants of the *TNF* gene promoter (rs361525, rs1800629, rs1799964, rs1799724) in 56 stable IIM patients using commercially available TaqMan allelic discrimination assays with real-time PCR. Clinical and laboratory data were collected to evaluate associations between genotypes and disease manifestations. Control subjects (n = 38) matched for sex and without a medical history of connective tissue diseases were included for comparison. The variant rs1800629 was the only one associated with increased susceptibility to the disease (p = 0.010), with a higher frequency of the AG genotype in the IIM group compared to controls. The same genotype (rs1800629 AG) was also associated with interstitial lung disease (ILD) in the patients (p = 0.024). Furthermore, ILD was related to the rs1799964 TT genotype (p = 0.049), which was also more frequent in those with anti-Jo1 antibodies (p = 0.012). The CT variant rs1799724 was associated with skin manifestations, such as the Gottron sign (p = 0.030) and the shawl sign (p = 0.021). No significant relationship was documented for the promoter variant of the rs361525 *TNF* gene. Our study revealed associations of specific variants of the *TNF* gene promoter with susceptibility to IIM and clinical manifestations of the disease. Extensive observational studies are needed to verify our findings.

**Supplementary Information:**

The online version contains supplementary material available at 10.1038/s41598-025-25258-z.

## Introduction

Idiopathic inflammatory myopathies (IIM) are a heterogeneous pathogenic group of diseases characterized by weakness of the proximal skeletal muscle and evidence of immune-mediated muscle injury^1^. Advances in IIM classification now incorporate clinical manifestations, histological findings, and autoantibody profiles, facilitating more nuanced differentiation between subtypes^2,3^. Adult cases are typically divided into dermatomyositis (DM), anti-synthetase syndrome-associated myositis, immune-mediated necrotizing myopathy (IMNM), inclusion body myositis (IBM), and unclassified polymyositis^4^. This modern approach underlines the role of myositis-specific antibodies (MSAs) in the prediction of the disease phenotype, including associated complications such as interstitial lung disease (ILD), skin changes, or Raynaud phenomenon^5,6^.

IIM have substantial diversity in clinical presentation, patterns of organ involvement, and natural history^7^. Some myositis-specific antibodies have been reported to be associated with certain clinical manifestations of the disease^5^. For example, anti-synthetase syndrome is characterized by antibodies directed against an aminoacyl transfer RNA synthetase and typical clinical features, ILD, myositis, Raynaud phenomenon, and arthritis^8^. In some cases, IIM are associated with other connective tissue disorders, such as systemic sclerosis (SSc), systemic lupus erythematosus, Sjögren syndrome, and rheumatoid arthritis^9^. In this situation, some authors particularize IIM overlap syndrome with a specific type of rheumatic disease, highlighting the complex nature of the pathology and clinics in this condition^10^.

The exact pathogenesis of IIM remains unknown, although some potential mechanisms have been proposed, including inflammatory cell infiltration^3^, MSAs-specific pathogenesis^11^, oxidative stress response^12^, endothelial dysfunction^13^, and thrombosis-related alteration^14^. All of them could be related to the overproduction of inflammatory cytokines, e.g., tumor necrosis factor (TNF), interleukin(IL)-1, IL-6, IL-17, and interferons (IFNs)^1,3,6^. IFNs and TNF are considered key pro-inflammatory cytokines in IIM, driving immune cell recruitment and muscle damage^15,16^. TNF is produced primarily by macrophages and is involved in all pathogenetic responses related to IIM, including muscle cell apoptosis and muscle fiber fibrosis, by stimulating fibroblast proliferation and collagen production^2,17,18^. TNF also activates the vascular endothelium, enhancing the expression of adhesion molecules that facilitate the recruitment and activation of inflammatory cells to the muscle^19,20^. Presented TNF-related mechanisms contribute to acute and chronic muscle damage; however, anti-TNF therapies, effective in other inflammatory and rheumatic diseases, have shown limited benefits in IIM^21,22^.

On the other hand, it has been documented that TNF is also involved in the development of malignancy, promoting inflammation, angiogenesis, and tumor evasion, which contribute to the progression of neoplastic diseases^23^. Interestingly, our previous study on patients with SSc revealed a link between increased serum TNF levels and the risk of malignancy or reduced survival in the long-extended follow-up, highlighting its dual role in inflammation and oncogenesis^24^. A similar observation has recently been made by Li et al.^25^, who demonstrated that TNF concentration could serve as a potential biomarker for cancer development among DM patients with anti-TIF1-γ antibodies. Interestingly, circulating TNF levels could be associated with specific *TNF* gene promoter variants, as previously documented in patients with SSc^24^.

In the past, several studies have investigated associations between IIM susceptibility and allelic *TNF* variants, particularly regarding promoter structure^26–28^. For instance, Chinoy et al.^26^ revealed that rs1800629 (TNF-308A) and rs1799964 (TNF-1031 T) alleles are risk factors for IIM. Next, a study by Hristova et al.^27^ in Bulgarian DM patients showed that rs1799964 (TNF -1031CC) genotype was found only among DM patients, and the rs1799964/rs1800630/rs1799724 /rs1800629/rs1800610 (TNF-1031C/-863C/-857C/-308G/ + 489G) haplotype was more frequent in DM women. The same was reported by Dourmishev et al.^28^. Furthermore, it has been demonstrated that specific *TNF* gene promoter variants can be associated with an increased risk of developing malignancy, including gastric cancer (rs1799724 [TNF-857] and rs1799964 [TNF-1031])^29^ or breast cancer (rs1800629 [TNF-308])^30^, but even in assessing prognosis, e.g. rs361525 in colorectal cancer^31^. Interestingly, previously, we have reported that C rs1799724 variant of the *TNF* gene was related to the SSc development, while CT rs1799964 and AG rs361525 genotypes were associated with cancer susceptibility in those patients^32^.

Given reported associations between *TNF* genetics and susceptibility to IIM^33^ or malignancy^34^, we sought to investigate *TNF* gene promoter variants (rs361525, rs1800629, rs1799964, and rs1799724) in a larger cohort of patients with IIM. We also examined their relationship with clinical disease manifestations, including the risk of neoplastic disorders.

## Patients and methods

### Characteristics of the participants

IIM patients (n = 56) were enrolled at the Department of Allergy and Clinical Immunology and the Department of Dermatology of the University Hospital in Kraków, Poland, between 2014 and 2020. Diagnosis of IIM was based on the 1975 Bohan and Peter diagnostic criteria^35,36^. Only patients who met the criteria for “definite” or “probable” IIM with clinically stable disease were included. In 2017, the European League Against Rheumatism/American College of Rheumatology (EULAR/ACR) published the new classification criteria for IIM^37^. According to them, we distinguished IIM patients into having dermatomyositis (DM), which consisted of DM with signs of muscle injury, amyopathic dermatomyositis, and hypomyopathic dermatomyositis, and a group of “other myositis”. The “other myositis” group comprised patients diagnosed with IMNM, antisynthetase-syndrome myositis, and other polymyositis. For simplicity, this group of different diagnoses is called “myositis” in the text. We did not enroll patients with IBM due to its unique clinical and pathological features^38^. Moreover, we specified a subgroup of cases in which the IIM was associated with other connective tissue diseases, including SSc, systemic lupus erythematosus, Sjögren syndrome, and rheumatoid arthritis. We particularized this subgroup as an “overlap syndrome”.

Among constitutional (general) symptoms, we included fever, malaise, and weight loss^39^. Next, below, we provided definitions for each DM skin manifestation based in the literature^40^. Gottron’s sign was defined as erythematous macules or patches over the extensor surfaces of elbows, knuckles, knees, and ankles. The heliotrope rash was diagnosed when periorbital erythema with edema, most often of the upper eyelids, was present. The shawl sign was defined as violaceous or erythematous macules and patches over posterior shoulders, neck, upper back, and possibly lateral upper arms. The V sign was defined as erythematous, confluent macules and patches over the lower anterior neck and upper chest. The holster signs were diagnosed if symmetric poikiloderma of hips and lateral thighs below the greater trochanter were observed. Mechanic’s hands were defined as hyperkeratotic, scaling, and fingers and/or palms fissuring. Raynaud phenomenon was defined as episodic vasospasm of fingers and toes in response to cold with tri-phasic color change.

Myositis-associated ILD was evaluated based on a chest computed tomography^41^. Muscle weakness, usually symmetric and progressive, was objectively diagnosed by qualified medical staff. Dysphagia was diagnosed based on clinical symptoms and/or imaging examinations (i.e., videofluoroscopy)^42^. Joint involvement was defined as joint pain with or without true arthritis.

The control group (n = 38, No. of male participants = 10 [26.3%]) comprised volunteers from hospital personnel with no personal or family history of connective tissue diseases. Control subjects were matched with the patient group with respect to sex (p = 0.81).

The Bioethics Committee of Jagiellonian University Medical College approved this study (No. KBET/235/B/2013, date on 26 September 2013). All procedures adhered to the ethical principles outlined in the Declaration of Helsinki. Each participant received detailed information about the study’s methods and safety procedures and then provided written informed consent before study enrollment.

### Laboratory analysis

Antinuclear antibodies (ANA) were evaluated using an indirect immunofluorescence assay on HEp-2 cell line slides (EUROIMMUN, Lübeck, Germany). They were positive if the titer was at least 1:160. We also performed immunoblot technique specific for autoantibodies against mitochondrial M2 (AMA M2), Mi-2-alpha, Mi-2-beta, Ku, PM-Scl 100, PM-Scl 75, Jo-1, SRP, PL-7, PL-12, EJ, OJ, PCNA, Ro-52, TIF1-gamma, MDA5, NXP2, SAE1, SSA, SSB, dsDNA, centromere B, histones, nucleosomes, RNA pol III, and DFS70 (EUROIMMUN, Lübeck, Germany).

### Genotyping

Blood samples were collected from all subjects of the study. The samples were kept at 4 °C until DNA extraction, performed within six hours after blood collection. DNA was extracted from peripheral leucocytes using DNAzol reagent (Invitrogen Life Technologies, Carlsbad, CA, USA). Four single nucleotide polymorphisms (SNPs) located in the promotor of the *TNFA* gene were analyzed, namely rs361525, rs1800629, rs1799964, and rs1799724. SNPs were genotyped with commercially available TaqMan allelic discrimination assays (Life Technologies) using the 7900 HT Real-time PCR System (Applied Biosystems, Foster City, CA, USA). A mix of unlabeled PCR primers and TaqMan MGB probes labeled with FAM or VIC dye were applied. The reaction was performed in a 10-μL solution that contained 0.5 μL of a 40 × oligonucleotides mix, 5 μL of a 2 × TaqMan Genotyping Master mix (Applied Biosystems, all), and 2 μL of 50 ng genomic DNA. PCR conditions were as follows: initial denaturation at 95 °C for 15 min; 40 cycles at 95 °C for 10 s and 60 °C for 45 s. Genotyping was performed on coded and blinded samples in the presence of negative ones (no DNA) included in each 96-well plate for quality control. The genotyping results were determined by using SDS 2.3 Allelic Discrimination Software (Applied Biosystems). The frequency of specific *TNFA* genetic variants was checked in the Genome Aggregation Database (GnomAD) and Trans-Omics for Precision Medicine (TOPMED).

### Statistical elaboration

Statistical analysis was conducted using Statistica 13.3 software (TIBCO Software Inc., Palo Alto, CA, USA). Comparisons of the SNPs frequency in the *TNF* gene in different manifestations of study groups were calculated according to the exact Fisher test or the Chi^2^ test. Haplotype analysis was not performed due to the small sample size. Data distribution was evaluated using the Shapiro–Wilk test. We presented continuous data as mean with standard deviation, and were compared with a Kruskal–Wallis test or a multiple repetition test. A two-sided p-value of below 0.05 was deemed statistically significant.

## Results

### Clinical characteristics

The study group consisted of 56 patients. In 16 cases, we diagnosed DM, in 30 myositis, and 10 patients represented overlap syndrome (8 associated with SSc, one with rheumatoid arthritis, and one with systemic lupus erythematosus). The studied subgroups did not differ in demographics (Table 1).

**Table 1:** Demographic and clinical characteristics of the whole studied group and subgroups.

Parameter	Study group n = 56	DM patients n = 16	Myositis patients n = 30	Patients with overlap syndrome n = 10	p-value DM vs. myositis vs. patients with overlap syndrome
Male sex, n (%)	16 (28.6%)	6 (37.5%)	8 (26.7%)	2 (20.0%)	0.67
Age, years	54.7 ± 13.7	54.0 ± 14.5	54.6 ± 13.0	56.0 ± 15.8	0.75
Age at time of diagnosis, years	50.9 ± 13.4	48.9 ± 13.4	51.6 ± 13.1	52.0 ± 15.5	0.31
BMI, kg/m^2^	27.6 ± 4.9	27.0 ± 4.8	28.3 ± 4.6	26.1 ± 6.0	0.37
Malignancy, n (%)	7 (12.5%)	1 (6.3%)	6 (20.0%)	0 (0.0%)	0.30

Categorical variables are presented as numbers with percentages, continuous variables are presented as mean with standard deviation. Abbreviations: DM – dermatomyositis, n – number, BMI – body mass index.

In Table 2, we present the autoantibody profile and clinical characteristics of IIM patients.

**Table 2:** A summary of the autoantibody profile and clinical characteristics of patients with idiopathic inflammatory myopathies.

Parameter	Study group n = 56
Antinuclear antibodies	
Presence of antinuclear antibodies, n (%)	56 (100.0%)
Anti-mitochondrial-M2 antibodies presence, n (%)	1 (1.8%)
Anti-Mi-2-alpha antibodies presence, n (%)	3 (5.4%)
Anti-Mi-2-beta antibodies presence, n (%)	2 (3.6%)
Anti-Ku antibodies presence, n (%)	6 (10.7%)
Anti-PM-Scl 100 antibodies presence, n (%)	7 (12.5%)
Anti-PM-Scl 75 antibodies presence, n (%)	8 (14.3%)
Anti-Jo1 antibodies presence, n (%)	14 (14.3%)
Anti-SRP antibodies presence, n (%)	3 (5.4%)
Anti-PL7 antibodies presence, n (%)	4 (7.1%)
Anti-PL12 antibodies presence, n (%)	6 (10.7%)
Anti-EJ antibodies presence, n (%)	1 (1.8%)
Anti-OJ antibodies presence, n (%)	2 (3.6%)
Anti-PCNA antibodies presence, n (%)	1 (1.8%)
Anti-Ro-52 antibodies presence, n (%)	23 (41.1%)
Anti-TIF1 gamma antibodies presence, n (%)	5 (8.9%)
Anti-MDA5 antibodies presence, n (%)	4 (7.1%)
Anti-NXP2 antibodies presence, n (%)	4 (7.1%)
Anti-SAE1 antibodies presence, n (%)	3 (5.4%)
Anti-DFS70 antibodies presence, n (%)	2 (3.6%)
Anti-dsDNA antibodies presence, n (%)	6 (10.7%)
Anti-SSA antibodies presence, n (%)	1 (1.8%)
Anti-SSB antibodies presence, n (%)	1 (1.8%)
Anti-centromere B antibodies presence, n (%)	2 (3.6%)
Anti-nucleosome antibodies presence, n (%)	1 (1.8%)
Anti-histone antibodies presence, n (%)	1 (1.8%)
Anti-RNA pol III antibodies presence, n (%)	1 (1.8%)
Organ involvement	
Interstitial lung disease, n (%)	31 (55.4%)
Lung fibrosis, n (%)	20 (35.7%)
Ground glass opacity, n (%)	27 (48.2%)
Raynaud’s phenomenon, n (%)	14 (25.0%)
Skin involvement, n (%)	32 (57.1%)
Mechanic’s hands, n (%)	11 (19.6%)
Gottron’s sign, n (%)	8 (14.3%)
Heliotrope rash, n (%)	8 (14.3%)
Shawl sign, n (%)	6 (10.7%)
Holster sign, n (%)	1 (1.8%)
V sign, n (%)	11 (19.6%)
General symptoms, n (%)	24 (42.9%)
Muscle weakness – shoulder girdle, n (%)	31 (55.4%)
Muscle weakness – hip girdle, n (%)	37 (66.1%)
General muscle weakness, n (%)	30 (53.6%)
Joints involvement, n (%)	31 (55.4%)
Dysphagia, n (%)	9 (16.1%)
Treatment characteristic	
Current steroids therapy, n (%)	13 (23.2%)
Immunosuppressive treatment, n (%)	37 (66.1%)
Comorbidities	
Hypertension, n (%)	24 (42.9%)
Diabetes mellitus, n (%)	13 (23.2%)
Hypercholesterolemia, n (%)	31 (55.4%)
Pulmonary artery hypertension, n (%)	8 (14.3%)
Ischemic heart disease, n (%)	8 (14.3%)

Categorical variables are presented as numbers with percentages. Abbreviations: n – number.

Antinuclear antibodies were detected in all patients, with anti-Ro-52 antibody being the most prevalent (n = 23, 41.1%), followed by anti-Jo1 (n = 14, 25.0%) and anti-PM-Scl 75 antibodies (n = 8, 14.3%). Regarding clinical manifestations, as expected, the most common were muscle weakness, particularly regarding the hip and shoulder girdle. Skin involvement was documented in all DM. Interstitial lung disease was prevalent in PM, reported in 23 (76.7%) of the cases. Among constitutional signs, general weakness was the most frequent at enrolment, while other disease manifestations, such as the Raynaud phenomenon and dysphagia, were less frequent (Table 2).

At the enrollment, 13 patients (23.2%) were treated with systemic glucocorticosteroids, and 37 individuals (66.1%) received immunosuppressive drugs, such as azathioprine, mycophenolate mofetil, cyclosporine, cyclophosphamide, rituximab.

Among comorbidities, hypercholesterolemia was the most frequent (n = 31, 55.4%). It is worth noting that in the analyzed group, 7 (12.5%) patients (1 DM and 6 PM) were diagnosed with malignancy, including papillary thyroid carcinoma with endometrial adenocarcinoma in one case, bladder papilloma, cervical squamous cell carcinoma, diffuse large B-cell lymphoma, ovarian cancer, breast cancer, and unknown type of malignancy in one patient. Regarding neoplastic disease prevalence, we did not find statistical differences between analyzed subgroups (Table 1).

### Distribution of TNF gene promoter variants in the study group and controls

The allele frequencies of the analyzed *TNF* promoter gene variants (rs361525, rs1800629, rs1799964, and rs1799724) are presented in Table 3. The rs1800629 AG genotype was associated with increased IIM susceptibility (p = 0.010), with a decreased prevalence of rs1800629 GG genotype in the patient group than in the controls. We did not find such associations for other analyzed variants, i.e., rs361525, rs1799964and rs1799724.

**Table 3:** Genotypes and allele frequencies of the *TNF* gene promoter variants in the study group and controls.

Single-nucleotide polymorphism	Genotypes	Patient group n = 56	Control group n = 38	p-value
rs361525	AA AG GG	0 (0.0%) 5 (8.9%) 51 (91.1%)	0 (0.0%) 2 (5.3%) 36 (94.7%)	0.70
rs1800629	AA AG GG	2 (3.6%) 24 (42.9%) 30 (53.6%)	1 (2.6%) 6 (15.8%) 31 (81.6%)	**0.010**
rs1799964	CC CT TT	3 (5.4%) 14 (25.0%) 39 (69.6%)	1 (2.6%) 8 (21.1%) 29 (76.3%)	0.74
rs1799724	CC CT TT	39 (69.6%) 16 (28.6%) 1 (1.8%)	34 (89.5%) 4 (10.5%) 0 (0.0%)	0.052

Categorical variables are presented as numbers with percentages. Statistically significant result is bolded. Statistical analysis was performed using the Chi^2^ test or Fisher’s exact test, where appropriate. Abbreviations: n – number.

### Distribution of the TNF gene promoter variants in the subgroup analysis

Interestingly, rs1800629 AG variant was associated with an increased susceptibility to myositis but not to DM (Table 4). This variant and the rs1799724 CT genotype were also more common in patients with overlap syndrome than in controls.

**Table 4:** Genotypes and allele frequencies of the *TNF* gene promoter variants in the analyzed subgroups and controls.

Single- nucleotide polymorphisms	Genotypes	DM patients n = 16	Myositis patients n = 30	Patients with overlap syndrome n = 10	Control group n = 38	p-value DM vs. controls	p-value myositis vs. controls	p-value patients with overlap syndrome vs. controls
rs361525	AA AG GG	0 (0.0%) 2 (12.5%) 14 (87.5%)	0 (0.0%) 2 (6.7%) 28 (93.3%)	0 (0.0%) 1 (10.0%) 9 (90.0%)	0 (0.0%) 2 (5.3%) 36 (94.7%)	0.57	> 0.99	0.51
rs1800629	AA AG GG	0 (0.0%) 4 (25.0%) 12 (75.0%)	2 (6.7%) 14 (46.7%) 14 (46.7%)	0 (0.0%) 6 (60.0%) 4 (40.0%)	1 (2.6%) 6 (15.8%) 31 (81.6%)	0.62	**0.005**	**0.016**
rs1799964	CC CT TT	2 (12.5%) 4 (25.0%) 10 (62.5%)	1 (3.0%) 8 (27.0%) 21 (70.0%)	0 (0.0%) 2 (20.0%) 8 (80.0%)	1 (2.6%) 8 (21.1%) 29 (76.3%)	0.28	0.89	> 0.99
rs1799724	CC CT TT	11 (68.8%) 4 (25.0%) 1 (6.3%)	23 (76.7%) 7 (23.3%) 0 (0.0%)	5 (50.0%) 5 (50.0%) 0 (0.0%)	34 (89.5%) 4 (10.5%) 0 (0.0%)	0.12	0.19	**0.012**

Categorical variables are presented as numbers with percentages. Statistically significant results are bolded. Statistical analysis was performed using the Chi^2^ test or Fisher’s exact test, where appropriate. Abbreviations: DM – dermatomyositis, n – number.

### TNF gene variants and clinical parameters in idiopathic inflammatory myopathy patients

Then, we investigated the relationship between various TNF gene promoter variants and clinical IIM parameters, including autoantibody profiles and organ involvement in the patient group. Detailed results for each allelic variant are provided in the Supplementary Materials: rs361525 (Table S1), rs1800629 (Table S2), rs1799964 (Table S3), and rs1799724 (Table S4).

Regarding the rs361525 variant, in terms of allele frequency, we did not find any significant relationship with clinics and ANA profile (Table S1). However, IIM patients with general symptoms had only the GG genotype (p = 0.06 in genotype frequency analysis).

The rs1800629 variant revealed several significant associations (Table S2). Firstly, among IIM patients, those with ILD had a higher frequency of the AG genotype, while the GG genotype was less common compared to IIM patients without ILD (p = 0.024 and p = 0.008, respectively). On the other hand, Gottron’s sign was reported only in IIM individuals with the GG genotype of this variant (p = 0.015). In terms of allele frequency, no significant differences were found in the rs1800629 variant with respect to the autoantibody profile (p > 0.05 for all ANA types).

The rs1799964 variant also showed several associations (Table S3). Among IIM patients, the TT genotype was more common in those with pulmonary manifestations, including lung fibrosis and ground glass opacity (p = 0.049 and p = 0.025, respectively). Notably, all 14 patients with anti-Jo1 antibodies had the TT rs1799964 genotype, resulting in significantly different genotype distribution between patients with and without these antibodies (p = 0.012). Interestingly, this variant was also associated with hypercholesterolemia, as the TT genotype was more prevalent in IIM patients with this condition (p = 0.024).

Finally, we analyzed the relationship of the rs1799724 *TNF* variant allele frequencies with IIM clinics and ANA profile (Table S4). We documented that the CC genotype showed a significant association with the Gottron’s sign (p = 0.030), and the CT genotype with the shawl sign (p = 0.021). No significant differences in allele frequency of the rs1799724 variant were observed in relation to the autoantibody profile (p > 0.05 for all ANA types).

Neither of the analyzed genetic variants was associated with the presence of neoplastic diseases (Tables S1-S4).

## Discussion

Our study demonstrates associations between specific *TNF* gene promoter variants and susceptibility to IIM development and clinical presentation. The rs1800629 AG variant was the only one found more frequently in the IIM group than controls. The same variant and the rs1799964 TT were related to lung involvement in the form of ILD. On the other hand, the rs1799724 CT variant was found to be more frequent in patients with skin manifestations such as Gottron’s sign and shawl sign. No significant associations were documented for the rs361525 *TNF* promoter variant.

Our cohort’s demographic was similar across the three subgroups (DM, myositis, and overlap syndrome), with no significant differences in age, sex distribution, or BMI, but also in malignancy prevalence. However, in the current literature, myositis is related to a higher risk of malignancies^43^. We did not find such an association, likely due to the subgroups’ limited number of cases. Next, autoantibodies were present in all patients, with anti-Ro-52 antibodies being the most frequent. That aligns with previous literature indicating that anti-Ro-52 is a common autoantibody in IIM, often associated with more severe disease outcomes^44^.

According to a study by Kao et al.^45^, TNF and its receptor are upregulated in DM and might contribute to muscle inflammation. Notably, the *TNF* encoding gene is located in the short arm of chromosome 6 in the major histocompatibility complex class III region. Most of its known genetic variants are in the promoter region. Thus, they might affect the level of TNF production and likely clinical manifestation of different inflammatory diseases, as demonstrated for the rs361525 and rs1800629 variants^46^. In our study, patients with general symptoms had only the GG genotype of the rs361525 variant. On the other hand, those with ILD and Gottron’s sign, had differences in rs1800629 genotype distribution. Nevertheless, the research results on that subject are ambiguous, indicating the complexity of genetic and environmental factors that may modulate TNF secretion by inflammatory cells.

Our analysis of *TNF* promoter variants revealed that the rs1800629 AG genotype was more common in the IIM group than in the controls, suggesting its potential role in disease pathogenesis. That result lines data documented by others, e.g., in adult myositis^47^ and juvenile DM regarding disease duration and TNF production^48^. Furthermore, in our study, significant associations between specific *TNF* gene promoter variants and IIM clinics underscore their potential to aid disease prediction and management. For instance, the rs1800629 AG genotype was more prevalent in patients with ILD, similar to the rs1799964 TT genotype, which was also more common in those with anti-Jo1 antibody and hypercholesterolemia. The available literature lacks data on associations between *TNF* gene variants and clinical manifestation of IIM. TNF can lead to fever, fatigue, weight loss, anorexia, and malaise. Therefore, specific variants of that gene might be associated with constitutional signs. In our study, the GG genotype of the rs361525 variant was the only genotype present in IIM patients with general symptoms. Yet, the reports of the influence of *TNF* polymorphism rs361525 on the transcriptional regulation of TNF have been conflicting^49,50^.

In the analyzed population, the TT rs1799964 genotype showed a higher prevalence in pulmonary manifestations, including lung fibrosis and ground glass opacity, as compared to other genotypes. That observation is novel and interesting, particularly since, according to some studies, TNF mRNA expression was reduced in individuals with this genotype^51,52^. Furthermore, in our data, the TT rs1799964 genotype was present in all patients with anti-Jo1 antibodies (n = 14), also associated with ILD leading to pulmonary fibrosis^53^. Several studies indicated an association of *TNF* variants with lung fibrosis, e.g., the A alleles of the rs361525 and rs1800629 variants in silicosis^54^, or the A allele of the rs1800629 variant in hypersensitivity pneumonitis^55^. Therefore, these findings underscore the potential significance of *TNF* promoter variants for clinical manifestations, including autoantibody profile in some inflammatory diseases. Further research is warranted to elucidate the underlying mechanisms and potential clinical implications.

Interestingly, several publications have documented a temporal association between anti-TNF therapy in autoimmune and chronic inflammatory disease patients and the new onset of IIM^56–61^. The exact reasons remain unclear, though several hypotheses have been suggested to explain the phenomenon. For instance, the cytokine-shift hypothesis proposes that suppressing TNF enhances type I IFN expression by changing the balance of inflammatory responses^59,62–64^. Thus, a rise in type I IFN, which is known to play a significant role in the development of DM, might contribute to the occurrence or worsening of IFN-related symptoms^59,65,66^. Alternatively, inhibition of TNF disrupts apoptosis, resulting in a higher autoantigen presentation and new autoantibodies production^59,67^. Interestingly, it is suggested that the effect of rs1800629 cannot be explained by TNF-α levels, as this SNP does not affect its mRNA or protein, indicating that the association may instead be with the neighboring LTA gene, which encodes lymphotoxin A and ends 1,200 nucleotides upstream^68^. However, further extended studies are needed to establish the role of TNF and anti-TNF therapy in IIM pathogenesis, making it a topic of ongoing investigation.

### Study limitations

Despite valuable insights from this study, several limitations should be acknowledged. The sample size of IIM patients and controls is relatively small, with a case-to-control ratio of approximately 3:2), which resulted from the recruitment process. Particularly, the subgroup analyses need to be interpreted with caution. Additionally, we focused on only four specific *TNF* genetic variants (rs361525, rs1800629, rs1799964, and rs1799724). Lastly, we did not measure TNF or other cytokine levels in serum related to the pathogenesis of IIM, which could provide valuable functional evaluation of the presented genetic variants. However, investigating associations of the clinical characteristics in this rare disease with specific *TNF* promoter genetic variants provides a new perception of disease pathogenesis.

## Conclusions

Our study highlights the complex interplay between clinical manifestations of IIM and specific genetic variants of *TNF* promoter. It revealed that the rs1800629 AG variant was the one associated with IIM susceptibility, while the rs1800629 AG and the rs1799964 TT were related to lung involvement. Moreover, the rs1799964 TT was related to lung fibrosis and anti-Jo1 antibodies. Finally, the rs1799724 variant was associated with skin changes; the CC genotype was more common in patients with the Gottron’s sign, and the CT genotype in those with the shawl sign. Further research is needed to explore the mechanistic background of the presented associations.

## Supplementary Information

Supplementary Information.

## Data Availability

The datasets used and analyzed during the current study are available from the corresponding author upon reasonable request.
