# Supplementary material for: Associations between TNF gene promoter variants (rs361525, rs1800629, rs1799964, rs1799724) and the clinical course of idiopathic inflammatory myopathies
Source: Sci Rep. 2025 Nov 21;15:41396. doi: 10.1038/s41598-025-25258-z (PMC12639174; doi:10.1038/s41598-025-25258-z)
Supplement: Supplementary file 1 — Supplementary Information. [file 41598_2025_25258_MOESM1_ESM.doc]

**Supplementary materials**

**Table S1.** The relationship between rs361525 genotypes and comorbidities, clinical and autoantibodies characteristics in the whole studied group.

| Characteristics (parameter) | Patients  n = 56 | | rs361525 genotypes | | | p-value |
| --- | --- | --- | --- | --- | --- | --- |
|  |  |  | AA  n = 0 | AG  n = 5 | GG  n = 51 |  |
| Comorbidities | | | | | | |
| Hypertension, n (%) | with | 24 | 0 (0.0%) | 3 (12.5%) | 21 (87.5%) | 0.64 |
|  | without | 32 | 0 (0.0%) | 2 (6.3%) | 30 (93.7%) |  |
| Diabetes mellitus, n (%) | with | 13 | 0 (0.0%) | 3 (23.1%) | 10 (76.9%) | 0.08 |
|  | without | 43 | 0 (0.0%) | 2 (4.7%) | 41 (95.3%) |  |
| Hypercholesterolemia, n (%) | with | 31 | 0 (0.0%) | 3 (9.7%) | 28 (90.3%) | 1.00 |
|  | without | 25 | 0 (0.0%) | 2 (8.0%) | 23 (92.0%) |  |
| Pulmonary artery hypertension, n (%) | with | 8 | 0 (0.0%) | 0 (0.0%) | 8 (100.0%) | 1.00 |
|  | without | 48 | 0 (0.0%) | 5 (10.4%) | 43 (89.6%) |  |
| Ischemic heart disease, n (%) | with | 8 | 0 (0.0%) | 1 (12.5%) | 7 (87.5%) | 0.55 |
|  | without | 48 | 0 (0.0%) | 4 (8.3%) | 44 (91.7%) |  |
| Clinical features | | | | | | |
| Interstitial lung disease, n (%) | with | 29 | 0 (0.0%) | 1 (3.4%) | 28 (96.6%) | 0.17 |
|  | without | 25 | 0 (0.0%) | 4 (16.0%) | 21 (84.0%) |  |
| Lung fibrosis, n (%) | with | 20 | 0 (0.0%) | 0 (0.0%) | 20 (100.0%) | 0.15 |
|  | without | 36 | 0 (0.0%) | 5 (13.9%) | 31 (86.1%) |  |
| Ground glass opacity, n (%) | with | 27 | 0 (0.0%) | 1 (3.7%) | 26 (96.3%) | 0.35 |
|  | without | 29 | 0 (0.0%) | 4 (13.8%) | 25 (86.2%) |  |
| Raynaud’s phenomenon, n (%) | with | 14 | 0 (0.0%) | 2 (14.3%) | 12 (85.7%) | 0.60 |
|  | without | 42 | 0 (0.0%) | 3 (7.1%) | 39 (92.9%) |  |
| Skin involvement, n (%) | with | 32 | 0 (0.0%) | 4 (12.5%) | 28 (87.5%) | 0.38 |
|  | without | 24 | 0 (0.0%) | 1 (4.2%) | 23 (95.8%) |  |
| Mechanic’s hands, n (%) | with | 11 | 0 (0.0%) | 2 (18.2%) | 9 (81.8%) | 0.25 |
|  | without | 45 | 0 (0.0%) | 3 (6.7%) | 42 (93.3%) |  |
| Gottron’s sign, n (%) | with | 8 | 0 (0.0%) | 0 (0.0%) | 8 (100.0%) | 1.00 |
|  | without | 48 | 0 (0.0%) | 5 (10.4%) | 43 (89.6%) |  |
| Heliotrope rash, n (%) | with | 8 | 0 (0.0%) | 2 (25.0%) | 6 (75.0%) | 0.14 |
|  | without | 48 | 0 (0.0%) | 3 (6.3%) | 45 (93.8%) |  |
| Shawl sign, n (%) | with | 6 | 0 (0.0%) | 0 (0.0%) | 6 (100.0%) | 1.00 |
|  | without | 50 | 0 (0.0%) | 5 (10.0%) | 45 (90.0%) |  |
| V sign, n (%) | with | 11 | 0 (0.0%) | 1 (9.1%) | 10 (90.9%) | 1.00 |
|  | without | 45 | 0 (0.0%) | 4 (8.9%) | 41 (91.1%) |  |
| General symptoms, n (%) | with | 24 | 0 (0.0%) | 0 (0.0%) | 24 (100.0%) | 0.06 |
|  | without | 32 | 0 (0.0%) | 5 (15.6%) | 27 (84.4%) |  |
| Muscle weakness – shoulder girdle, n (%) | with | 31 | 0 (0.0%) | 2 (6.5%) | 29 (93.5%) | 0.65 |
|  | without | 25 | 0 (0.0%) | 3 (12.0%) | 22 (88.0%) |  |
| Muscle weakness – hip girdle, n (%) | with | 37 | 0 (0.0%) | 2 (5.4%) | 35 (94.6%) | 0.32 |
|  | without | 19 | 0 (0.0%) | 3 (15.8%) | 16 (84.2%) |  |
| General muscle weakness, n (%) | with | 30 | 0 (0.0%) | 4 (13.3%) | 26 (86.7%) | 0.36 |
|  | without | 26 | 0 (0.0%) | 1 (3.8%) | 25 (96.2%) |  |
| Joints involvement, n (%) | with | 31 | 0 (0.0%) | 2 (6.5%) | 29 (93.5%) | 0.65 |
|  | without | 25 | 0 (0.0%) | 3 (12.0%) | 22 (88.0%) |  |
| Dysphagia, n (%) | with | 9 | 0 (0.0%) | 2 (22.2%) | 7 (77.8%) | 0.18 |
|  | without | 47 | 0 (0.0%) | 3 (6.4%) | 44 (93.6%) |  |
| Antinuclear antibodies | | | | | | |
| Anti-Ku antibodies presence, n (%) | with | 6 | 0 (0.0%) | 1 (16.7%) | 5 (83.3%) | 0.45 |
|  | without | 50 | 0 (0.0%) | 4 (8.0%) | 46 (92.0%) |  |
| Anti-PM-Scl 100 antibodies presence, n (%) | with | 7 | 0 (0.0%) | 1 (14.3%) | 6 (85.7%) | 0.50 |
|  | without | 49 | 0 (0.0%) | 4 (8.2%) | 45 (91.8%) |  |
| Anti-PM-Scl 75 antibodies presence, n (%) | with | 8 | 0 (0.0%) | 1 (12.5%) | 7 (87.5%) | 0.55 |
|  | without | 48 | 0 (0.0%) | 4 (8.3%) | 44 (91.7%) |  |
| Anti-Jo1 antibodies presence, n (%) | with | 14 | 0 (0.0%) | 0 (0.0%) | 14 (100.0%) | 0.32 |
|  | without | 42 | 0 (0.0%) | 5 (11.9%) | 37 (88.1%) |  |
| Anti-PL12 antibodies presence, n (%) | with | 6 | 0 (0.0%) | 0 (0.0%) | 6 (100.0%) | 1.00 |
|  | without | 50 | 0 (0.0%) | 5 (10.0%) | 45 (90.0%) |  |
| Anti-Ro-52 antibodies presence, n (%) | with | 23 | 0 (0.0%) | 2 (8.7%) | 21 (91.3%) | 1.00 |
|  | without | 33 | 0 (0.0%) | 3 (9.1%) | 30 (90.9%) |  |
| Anti-dsDNA antibodies presence, n (%) | with | 6 | 0 (0.0%) | 0 (0.0%) | 6 (100.0%) | 1.00 |
|  | without | 50 | 0 (0.0%) | 5 (10.0%) | 45 (90.0%) |  |

Categorical variables are presented as numbers with percentages. Statistically significant results are bolded. Abbreviations: n – number.

**Table S2.** The relationship between rs1800629 genotypes and comorbidities, clinical and autoantibodies characteristics in the whole studied group.

| Characteristics (parameter) | Patients  n = 56 | | rs1800629 genotypes | | | p-value |
| --- | --- | --- | --- | --- | --- | --- |
|  |  |  | AA  n = 2 | AG  n = 24 | GG  n = 30 |  |
| Comorbidities | | | | | | |
| Hypertension, n (%) | with | 24 | 0 (0.0%) | 13 (54.2%) | 11 (45.8%) | 0.24 |
|  | without | 32 | 2 (6.3%) | 11 (34.4%) | 19 (59.4%) |  |
| Diabetes mellitus, n (%) | with | 13 | 0 (0.0%) | 3 (23.1%) | 10 (76.9%) | 0.18 |
|  | without | 43 | 2 (4.7%) | 21 (48.8%) | 20 (46.5%) |  |
| Hypercholesterolemia, n (%) | with | 31 | 0 (0.0%) | 16 (51.6%) | 15 (48.4%) | 0.13 |
|  | without | 25 | 2 (8.0%) | 8 (32.0%) | 15 (60.0%) |  |
| Pulmonary artery hypertension, n (%) | with | 8 | 0 (0.0%) | 5 (62.5%) | 3 (37.5%) | 0.59 |
|  | without | 48 | 2 (4.2%) | 19 (39.6%) | 27 (56.3%) |  |
| Ischemic heart disease, n (%) | with | 8 | 0 (0.0%) | 2 (25.0%) | 6 (75.0%) | 0.47 |
|  | without | 48 | 2 (4.2%) | 22 (45.8%) | 24 (50.0%) |  |
| Clinical features | | | | | | |
| Interstitial lung disease, n (%) | with | 29 | 0 (0.0%) | 17 (58.6%) | 12 (41.4%) | **0.024** |
|  | without | 25 | 2 (8.0%) | 7 (28.0%) | 16 (64.0%) |  |
| Lung fibrosis, n (%) | with | 20 | 0 (0.0%) | 12 (60.0%) | 8 (40.0%) | 0.13 |
|  | without | 36 | 2 (5.6%) | 12 (33.3%) | 22 (61.1%) |  |
| Ground glass opacity, n (%) | with | 27 | 0 (0.0%) | 17 (63.0%) | 10 (37.0%) | **0.008** |
|  | without | 29 | 2 (6.9%) | 7 (24.1%) | 20 (69.0%) |  |
| Raynaud’s phenomenon, n (%) | with | 14 | 2 (14.3%) | 5 (35.7%) | 7 (50.0%) | 0.10 |
|  | without | 42 | 0 (0.0%) | 19 (45.2%) | 23 (54.8%) |  |
| Skin involvement, n (%) | with | 32 | 0 (0.0%) | 13 (40.6%) | 19 (59.4%) | 0.24 |
|  | without | 24 | 2 (8.3%) | 11 (45.8%) | 11 (45.8%) |  |
| Mechanic’s hands, n (%) | with | 11 | 0 (0.0%) | 7 (63.6%) | 4 (36.4%) | 0.31 |
|  | without | 45 | 2 (4.4%) | 17 (37.8%) | 26 (57.8%) |  |
| Gottron’s sign, n (%) | with | 8 | 0 (0.0%) | 0 (0.0%) | 8 (100.0%) | **0.015** |
|  | without | 48 | 2 (4.2%) | 24 (50.0%) | 22 (45.8%) |  |
| Heliotrope rash, n (%) | with | 8 | 0 (0.0%) | 3 (37.5%) | 5 (62.5%) | 0.80 |
|  | without | 48 | 2 (4.2%) | 21 (43.8%) | 25 (52.1%) |  |
| Shawl sign, n (%) | with | 6 | 0 (0.0%) | 1 (16.7%) | 5 (83.3%) | 0.37 |
|  | without | 50 | 2 (4.0%) | 23 (46.0%) | 25 (50.0%) |  |
| V sign, n (%) | with | 11 | 0 (0.0%) | 3 (27.3%) | 8 (72.7%) | 0.39 |
|  | without | 45 | 2 (4.4%) | 21 (46.7%) | 22 (48.9%) |  |
| General symptoms, n (%) | with | 24 | 0 (0.0%) | 11 (45.8%) | 13 (54.2%) | 0.71 |
|  | without | 32 | 2 (6.3%) | 13 (40.6%) | 17 (53.1%) |  |
| Muscle weakness – shoulder girdle, n (%) | with | 31 | 0 (0.0%) | 13 (41.9%) | 18 (58.1%) | 0.36 |
|  | without | 25 | 2 (8.0%) | 11 (4.0%) | 12 (48.0%) |  |
| Muscle weakness – hip girdle, n (%) | with | 37 | 2 (5.4%) | 14 (37.8%) | 21 (56.8%) | 0.46 |
|  | without | 19 | 0 (0.0%) | 10 (52.6%) | 9 (47.4%) |  |
| General muscle weakness, n (%) | with | 30 | 1 (3.3%) | 11 (36.7%) | 18 (60.0%) | 0.70 |
|  | without | 26 | 1 (3.8%) | 13 (50.0%) | 12 (46.2%) |  |
| Joints involvement, n (%) | with | 31 | 2 (6.5%) | 15 (48.4%) | 14 (45.2%) | 0.25 |
|  | without | 25 | 0 (0.0%) | 9 (36.0%) | 16 (64.0%) |  |
| Dysphagia, n (%) | with | 9 | 0 (0.0%) | 5 (55.6%) | 4 (44.4%) | 0.64 |
|  | without | 47 | 2 (4.3%) | 19 (40.4%) | 26 (55.3%) |  |
| Antinuclear antibodies | | | | | | |
| Anti-Ku antibodies presence, n (%) | with | 6 | 0 (0.0%) | 1 (16.7%) | 5 (83.3%) | 0.37 |
|  | without | 50 | 2 (4.0%) | 23 (46.0%) | 25 (50.0%) |  |
| Anti-PM-Scl 100 antibodies presence, n (%) | with | 7 | 0 (0.0%) | 3 (42.9%) | 4 (57.1%) | 1.00 |
|  | without | 49 | 2 (4.1%) | 21 (42.9%) | 26 (53.1%) |  |
| Anti-PM-Scl 75 antibodies presence, n (%) | with | 8 | 0 (0.0%) | 2 (25.0%) | 6 (75.0%) | 0.47 |
|  | without | 48 | 2 (4.2%) | 22 (45.8%) | 24 (50.0%) |  |
| Anti-Jo1 antibodies presence, n (%) | with | 14 | 1 (7.1%) | 8 (57.1%) | 5 (35.7%) | 0.20 |
|  | without | 42 | 1 (2.4%) | 16 (38.1%) | 25 (59.5%) |  |
| Anti-PL12 antibodies presence, n (%) | with | 6 | 0 (0.0%) | 3 (50.0%) | 3 (50.0%) | 1.00 |
|  | without | 50 | 2 (4.0%) | 21 (42.0%) | 27 (54.0%) |  |
| Anti-Ro-52 antibodies presence, n (%) | with | 23 | 0 (0.0%) | 13 (56.5%) | 10 (43.5%) | 0.17 |
|  | without | 33 | 2 (6.1%) | 11 (33.3%) | 20 (60.6%) |  |
| Anti-dsDNA antibodies presence, n (%) | with | 6 | 0 (0.0%) | 3 (50.0%) | 3 (50.0%) | 1.00 |
|  | without | 50 | 2 (4.0%) | 21 (42.0%) | 27 (54.0%) |  |

Categorical variables are presented as numbers with percentages. Statistically significant results are bolded. Abbreviations: n – number.

**Table S3.** The relationship between rs1799964 genotypes and comorbidities, clinical and autoantibodies characteristics in the whole studied group.

| Characteristics (parameter) | Patients  n = 56 | | rs1799964 genotypes | | | p-value |
| --- | --- | --- | --- | --- | --- | --- |
|  |  |  | CC  n = 3 | CT  n = 14 | TT  n = 39 |  |
| Comorbidities | | | | | | |
| Hypertension, n (%) | with | 24 | 1 (4.2%) | 7 (29.2%) | 16 (66.7%) | 0.89 |
|  | without | 32 | 2 (6.3%) | 7 (21.9%) | 23 (71.9%) |  |
| Diabetes mellitus, n (%) | with | 13 | 1 (7.7%) | 5 (38.5%) | 7 (53.8%) | 0.31 |
|  | without | 43 | 2 (4.7%) | 9 (20.9%) | 32 (74.4%) |  |
| Hypercholesterolemia, n (%) | with | 31 | 1 (3.2%) | 4 (12.9%) | 26 (83.9%) | **0.024** |
|  | without | 25 | 2 (8.0%) | 10 (40.0%) | 13 (52.0%) |  |
| Pulmonary artery hypertension, n (%) | with | 8 | 0 (0.0%) | 2 (25.0%) | 6 (75.0%) | 1.00 |
|  | without | 48 | 3 (6.3%) | 12 (25.0%) | 33 (68.8%) |  |
| Ischemic heart disease, n (%) | with | 8 | 0 (0.0%) | 3 (37.5%) | 5 (62.5%) | 0.64 |
|  | without | 48 | 3 (6.3%) | 11 (22.9%) | 34 (70.8%) |  |
| Clinical features | | | | | | |
| Interstitial lung disease, n (%) | with | 31 | 2 (6.5%) | 6 (19.4%) | 23 (74.2%) | 0.51 |
|  | without | 25 | 1 (4.0%) | 8 (32.0%) | 16 (64.0%) |  |
| Lung fibrosis, n (%) | with | 20 | 0 (0.0%) | 2 (10.0%) | 18 (90.0%) | **0.049** |
|  | without | 36 | 3 (8.3%) | 12 (33.3%) | 21 (58.3%) |  |
| Ground glass opacity, n (%) | with | 27 | 0 (0.0%) | 4 (14.8%) | 23 (85.2%) | **0.025** |
|  | without | 29 | 3 (10.3%) | 10 (34.5%) | 16 (55.2%) |  |
| Raynaud’s phenomenon, n (%) | with | 14 | 1 (7.1%) | 5 (35.7%) | 8 (57.1%) | 0.40 |
|  | without | 42 | 2 (4.8%) | 9 (21.4%) | 31 (73.8%) |  |
| Skin involvement, n (%) | with | 32 | 2 (6.3%) | 10 (31.3%) | 20 (62.5%) | 0.46 |
|  | without | 24 | 1 (4.2%) | 4 (16.7%) | 19 (79.2%) |  |
| Mechanic’s hands, n (%) | with | 11 | 0 (0.0%) | 3 (27.3%) | 8 (72.7%) | 1.00 |
|  | without | 45 | 3 (6.7%) | 11 (24.4%) | 31 (68.9%) |  |
| Gottron’s sign, n (%) | with | 8 | 1 (12.5%) | 1 (12.5%) | 6 (75.0%) | 0.38 |
|  | without | 48 | 2 (4.2%) | 13 (27.1%) | 33 (68.8%) |  |
| Heliotrope rash, n (%) | with | 8 | 0 (0.0%) | 4 (50.0%) | 4 (50.0%) | 0.22 |
|  | without | 48 | 3 (6.3%) | 10 (20.8%) | 35 (72.9%) |  |
| Shawl sign, n (%) | with | 6 | 0 (0.0%) | 0 (0.0%) | 6 (100.0%) | 0.31 |
|  | without | 50 | 3 (6.0%) | 14 (28.0%) | 33 (66.0%) |  |
| V sign, n (%) | with | 11 | 0 (0.0%) | 3 (27.3%) | 8 (72.7%) | 1.00 |
|  | without | 45 | 3 (6.7%) | 11 (24.4%) | 31 (68.9%) |  |
| General symptoms, n (%) | with | 24 | 1 (4.2%) | 4 (16.7%) | 19 (79.2%) | 0.46 |
|  | without | 32 | 2 (6.3%) | 10 (31.3%) | 20 (62.5%) |  |
| Muscle weakness – shoulder girdle, n (%) | with | 31 | 2 (6.5%) | 6 (19.4%) | 23 (74.2%) | 0.51 |
|  | without | 25 | 1 (4.0%) | 8 (32.0%) | 16 (64.0%) |  |
| Muscle weakness – hip girdle, n (%) | with | 37 | 2 (5.4%) | 6 (16.2%) | 29 (78.4%) | 0.10 |
|  | without | 19 | 1 (5.3%) | 8 (42.1%) | 10 (52.6%) |  |
| General muscle weakness, n (%) | with | 30 | 2 (6.7%) | 7 (23.3%) | 21 (70.0%) | 1.00 |
|  | without | 26 | 1 (3.8%) | 7 (26.9%) | 18 (69.2%) |  |
| Joints involvement, n (%) | with | 31 | 1 (3.2%) | 5 (16.1%) | 25 (80.6%) | 0.14 |
|  | without | 25 | 2 (8.0%) | 9 (36.0%) | 14 (56.0%) |  |
| Dysphagia, n (%) | with | 9 | 1 (11.1%) | 2 (22.2%) | 6 (66.7%) | 0.66 |
|  | without | 47 | 2 (4.3%) | 12 (25.5%) | 33 (70.2%) |  |
| Antinuclear antibodies | | | | | | |
| Anti-Ku antibodies presence, n (%) | with | 6 | 1 (16.7%) | 1 (16.7%) | 4 (66.7%) | 0.52 |
|  | without | 50 | 2 (4.0%) | 13 (26.0%) | 35 (70.0%) |  |
| Anti-PM-Scl 100 antibodies presence, n (%) | with | 7 | 0 (0.0%) | 2 (28.6%) | 5 (71.4%) | 1.00 |
|  | without | 49 | 3 (6.1%) | 12 (24.5%) | 34 (69.4%) |  |
| Anti-PM-Scl 75 antibodies presence, n (%) | with | 8 | 0 (0.0%) | 2 (25.0%) | 6 (75.0%) | 1.00 |
|  | without | 48 | 3 (6.3%) | 12 (25.0%) | 33 (68.8%) |  |
| Anti-Jo1 antibodies presence, n (%) | with | 14 | 0 (0.0%) | 0 (0.0%) | 14 (100.0%) | **0.012** |
|  | without | 42 | 3 (7.1%) | 14 (33.3%) | 25 (59.5%) |  |
| Anti-PL12 antibodies presence, n (%) | with | 6 | 0 (0.0%) | 2 (33.3%) | 4 (66.7%) | 0.75 |
|  | without | 50 | 3 (6.0%) | 12 (24.0%) | 35 (70.0%) |  |
| Anti-Ro-52 antibodies presence, n (%) | with | 23 | 1 (4.3%) | 4 (17.4%) | 18 (78.3%) | 0.57 |
|  | without | 33 | 2 (6.1%) | 10 (30.3%) | 21 (63.6%) |  |
| Anti-dsDNA antibodies presence, n (%) | with | 6 | 0 (0.0%) | 0 (0.0%) | 6 (100.0%) | 0.31 |
|  | without | 50 | 3 (6.0%) | 14 (28.0%) | 33 (66.0%) |  |

Categorical variables are presented as numbers with percentages. Statistically significant results are bolded. Abbreviations: n – number.

**Table S4.** The relationship between rs1799724 genotypes and comorbidities, clinical and autoantibodies characteristics in the whole studied group.

| Characteristics (parameter) | Patients  n = 56 | | rs1799724 genotypes | | | p-value |
| --- | --- | --- | --- | --- | --- | --- |
|  |  |  | CC  n = 39 | CT  n = 16 | TT  n = 1 |  |
| Comorbidities | | | | | | |
| Hypertension, n (%) | with | 24 | 18 (75.0%) | 6 (25.0%) | 0 (0.0%) | 0.87 |
|  | without | 32 | 21 (65.6%) | 10 (31.3%) | 1 (3.1%) |  |
| Diabetes mellitus, n (%) | with | 13 | 8 (61.5%) | 5 (38.5%) | 0 (0.0%) | 0.61 |
|  | without | 43 | 31 (72.1%) | 11 (25.6%) | 1 (2.3%) |  |
| Hypercholesterolemia, n (%) | with | 31 | 21 (67.7%) | 10 (32.3%) | 0 (0.0%) | 0.55 |
|  | without | 25 | 18 (72.0%) | 6 (24.0%) | 1 (4.0%) |  |
| Pulmonary artery hypertension, n (%) | with | 8 | 6 (75.0%) | 2 (25.0%) | 0 (0.0%) | 1.00 |
|  | without | 48 | 33 (68.8%) | 14 (29.2%) | 1 (2.1%) |  |
| Ischemic heart disease, n (%) | with | 8 | 5 (62.5%) | 3 (37.5%) | 0 (0.0%) | 0.72 |
|  | without | 48 | 34 (70.8%) | 13 (27.1%) | 1 (2.1%) |  |
| Clinical features | | | | | | |
| Interstitial lung disease, n (%) | with | 29 | 19 (65.5%) | 9 (31.0%) | 1 (3.4%) | 0.77 |
|  | without | 25 | 18 (74.1%) | 7 (25.9%) | 0 (0.0% |  |
| Lung fibrosis, n (%) | with | 20 | 12 (60.0%) | 7 (35.0%) | 1 (5.0%) | 0.21 |
|  | without | 36 | 27 (75.0%) | 9 (25.0%) | 0 (0.0%) |  |
| Ground glass opacity, n (%) | with | 27 | 18 (66.7%) | 8 (29.6%) | 1 (3.7%) | 0.88 |
|  | without | 29 | 21 (72.4%) | 8 (7.6%) | 0 (0.0%) |  |
| Raynaud’s phenomenon, n (%) | with | 14 | 10 (71.4%) | 4 (28.6%) | 0 (0.0%) | 1.00 |
|  | without | 42 | 29 (69.0%) | 12 (28.6%) | 1 (2.4%) |  |
| Skin involvement, n (%) | with | 32 | 20 (62.5%) | 11 (34.4%) | 1 (3.1%) | 0.37 |
|  | without | 24 | 19 (79.2%) | 5 (20.8%) | 0 (0.0%) |  |
| Mechanic’s hands, n (%) | with | 11 | 7 (63.6%) | 4 (36.4%) | 0 (0.0%) | 0.77 |
|  | without | 45 | 32 (71.1%) | 12 (26.7%) | 1 (2.2%) |  |
| Gottron’s sign, n (%) | with | 8 | 3 (37.5%) | 4 (50.0%) | 1 (12.5%) | **0.030** |
|  | without | 48 | 36 (75.0%) | 12 (25.0%) | 0 (0.0%) |  |
| Heliotrope rash, n (%) | with | 8 | 6 (75.0%) | 1 (12.5%) | 1 (12.5%) | 0.13 |
|  | without | 48 | 33 (68.8%) | 15 (31.3%) | 0 (0.0%) |  |
| Shawl sign, n (%) | with | 6 | 2 (33.3%) | 3 (50.0%) | 1 (16.7%) | **0.021** |
|  | without | 50 | 37 (74.0%) | 13 (26.0%) | 0 (0.0%) |  |
| V sign, n (%) | with | 11 | 8 (72.7%) | 2 (18.2%) | 1 (9.1%) | 0.19 |
|  | without | 45 | 31 (68.9%) | 14 (31.1%) | 0 (0.0%) |  |
| General symptoms, n (%) | with | 24 | 18 (75.0%) | 5 (20.8%) | 1 (4.2%) | 0.24 |
|  | without | 32 | 21 (65.6%) | 11 (34.4%) | 0 (0.0%) |  |
| Muscle weakness – shoulder girdle, n (%) | with | 31 | 19 (61.3%) | 11 (35.5%) | 1 (3.2%) | 0.30 |
|  | without | 25 | 20 (80.0%) | 5 (20.0%) | 0 (0.0%) |  |
| Muscle weakness – hip girdle, n (%) | with | 37 | 23 (62.2%) | 13 (35.1%) | 1 (2.7%) | 0.21 |
|  | without | 19 | 16 (84.2%) | 3 (15.8%) | 0 (0.0%) |  |
| General muscle weakness, n (%) | with | 30 | 22 (73.3%) | 7 (23.3%) | 1 (3.3%) | 0.55 |
|  | without | 26 | 17 (65.4%) | 9 (34.6%) | 0 (0.0%) |  |
| Joints involvement, n (%) | with | 31 | 23 (74.2%) | 8 (25.8%) | 0 (0.0%) | 0.46 |
|  | without | 25 | 16 (64.0%) | 8 (32.0%) | 1 (4.0%) |  |
| Dysphagia, n (%) | with | 9 | 7 (77.8%) | 1 (11.1%) | 1 (11.1%) | 0.11 |
|  | without | 47 | 32 (68.1%) | 15 (31.9%) | 0 (0.0%) |  |
| Antinuclear antibodies | | | | | | |
| Anti-Ku antibodies presence, n (%) | with | 3 | 3 (100.0%) | 0 (0.0%) | 0 (0.0%) | 0.57 |
|  | without | 50 | 32 (66.0%) | 16 (32.0%) | 1 (2.0%) |  |
| Anti-PM-Scl 100 antibodies presence, n (%) | with | 6 | 6 (100.0%) | 0 (0.0%) | 0 (0.0%) | 0.25 |
|  | without | 49 | 32 (65.3%) | 16 (32.7%) | 1 (2.0%) |  |
| Anti-PM-Scl 75 antibodies presence, n (%) | with | 8 | 5 (62.5%) | 3 (37.5%) | 0 (0.0%) | 0.72 |
|  | without | 48 | 34 (70.8%) | 13 (27.1%) | 1 (2.1%) |  |
| Anti-Jo1 antibodies presence, n (%) | with | 14 | 10 (71.4%) | 4 (28.6%) | 0 (0.0%) | 1.00 |
|  | without | 42 | 29 (69.0%) | 12 (28.6%) | 1 (2.4%) |  |
| Anti-PL12 antibodies presence, n (%) | with | 6 | 4 (66.7%) | 2 (33.3%) | 0 (0.0%) | 1.00 |
|  | without | 50 | 35 (70.0%) | 14 (28.0%) | 1 (2.0%) |  |
| Anti-Ro-52 antibodies presence, n (%) | with | 23 | 16 (69.6%) | 6 (26.1%) | 1 (4.3%) | 0.63 |
|  | without | 33 | 23 (69.7%) | 10 (30.3%) | 0 (0.0%) |  |
| Anti-dsDNA antibodies presence, n (%) | with | 6 | 3 (50.0%) | 3 (50.0%) | 0 (0.0%) | 0.41 |
|  | without | 50 | 36 (72.0%) | 13 (26.0%) | 1 (2.0%) |  |

Categorical variables are presented as numbers with percentages. Statistically significant results are bolded. Abbreviations: n – number.
